# Study on thermal adaptation behaviors of bus passengers

**DOI:** 10.3389/fpubh.2025.1707181

**Published:** 2025-11-28

**Authors:** Tao Zou, Xiaofang Yang, Yixuan Liu, Yuhui Wang, Fangyuan Zheng, Yifan Zhao, Yanlin Bao

**Affiliations:** 1 School of Architecture and Art, Central South University, Changsha, China; 2 Lushan Laboratory, Changsha, Hunan, China; 3 Bartlett School of Architecture, University College London (UCL), London, United Kingdom

**Keywords:** bus thermal comfort, thermal adaptation, passenger behavior, structural equation modeling, integrated mode

## Abstract

Thermal comfort (TC) inside buses has always been a key consideration for passengers when traveling. Although numerous studies and policies have explored and guided air conditioning standards for buses, research on passengers' thermal adaptation behaviors remains scarce—particularly in the context of widespread dissatisfaction with the thermal environment on buses. In this study, we adopted a mixed-methods approach and developed a new integrated research model to investigate passengers' thermal adaptation behaviors and their influencing factors. By constructing a theoretical framework for bus passengers' thermal adaptation behaviors, this study provides theoretical support for enhancing thermal comfort evaluation and deepening the understanding of the mechanisms underlying passengers' thermal adaptation behaviors. The results identified perceived behavioral control (PBC) and thermal environment attitude as the most substantial positive predictors of adaptation intention. The findings provide practical insights for improving the thermal comfort design of buses, enhancing passengers' satisfaction during transit, and guiding manufacturers and service providers in optimizing bus production and services.

## 1 Introduction

The transportation industry is at a historic turning point, with the concept of mobility undergoing a paradigm shift (1). Increasing attention is being paid to creating a comfortable travel environment, particularly focusing on thermal comfort (TC) within vehicles (2). As of 2022, over 700,000 buses operate across China's prefecture-level cities, with around 540,000 of these being new energy buses. Urban buses offer the advantage of transporting large numbers of passengers, making them a sustainable mode of transportation (3). However, this also places higher demands on the quality of the bus environment. The onboard environment affects passengers' perceptions of service quality and their willingness to choose this form of transportation (4). Among the various aspects influencing the bus environment, thermal comfort is considered one of the key factors affecting passenger comfort (5).

Thermal comfort is defined by ASHRAE (the American Society of Heating, Refrigerating, and Air-Conditioning Engineers) as a mental state of satisfaction with the thermal environment. Fanger's Predicted Mean Vote model is a traditional basis for assessing this, comprehensively evaluating the environment from physical, physiological, and psychological aspects (6). Passenger thermal comfort in vehicles is primarily influenced by heat exchange between the body and the internal compartment environment (7). Key factors affecting it include air temperature, humidity, mean radiant temperature, relative air velocity, human activity level, and clothing insulation (2). However, many complaints about bus thermal environments indicate existing AC and ventilation systems are inadequate (8, 9). For instance, interior-exterior temperature differences and poor ventilation cause discomfort, negatively impacting passenger experience and health (10).

An uncomfortable thermal environment inside the bus negatively affects both passenger physiology and psychology (11). Physiologically, it can cause symptoms such as dizziness, nausea, and fatigue, significantly impairing the riding experience (12). Psychologically, this discomfort may lead to emotional responses like irritability and anxiety, potentially reducing willingness to use bus services (13).

The thermal environment significantly impacts passenger willingness to ride and overall experience. When the thermal environment becomes uncomfortable, passengers engage in thermal adaptation behavior, defined as adaptive adjustments aimed at restoring thermal balance. This aligns with Humphreys' adaptation principle, which posits that individuals take behavioral actions in response to uncomfortable environmental changes (14). de Dear and Brager (8) classify thermal adaptation into three types: physiological adaptation, psychological acclimatization, and behavioral adjustments. Physiological adaptation involves the body's thermoregulatory responses (15). Psychological acclimatization reflects long-term adaptation and experience, enabling individuals to adjust their perception and reaction to temperature changes (16, 17). Thermal regulatory behaviors are influenced by physiological and psychological systems. Passengers primarily make behavioral adjustments when they reach their tolerance threshold of feeling too hot or too cold, adapting to the bus's thermal environment.

Passengers' thermal adaptation behaviors help them achieve a comfortable thermal environment, with appropriate thermal adaptation reducing health issues. Mylonas et al. (18) defines occupant behaviors based on factors such as activities and preferences, including actions like opening windows, turning lights on and off, and using heating, ventilation, and other equipment. However, the bus environment differs from building interiors; the cabin climate is mainly dominated by transient thermal conditions. Over 85% of bus rides cover distances under 18 km, with trip durations between 15 and 30 min (2). During their rides, passengers often sit or stand for extended periods, exposed to transient and non-uniform thermal conditions (19). Previous research indicates that thermal discomfort levels in transportation are notably higher than in buildings, and with high passenger density, the main ventilation system fails to meet individual needs. Common thermal adaptation behaviors on buses include opening or closing windows, adjusting the air conditioning, repositioning curtains, and adjusting clothing (e.g., putting on or taking off layers) (20). These actions are crucial for passengers to create a favorable personal thermal environment (21).

Although extensive research has been conducted on air conditioning standards, passenger-centered thermal adaptation behaviors remain underexplored. Passengers' individual perceptions play a key role in thermal adaptation behaviors on buses. This perception not only includes immediate reactions to the thermal environment but also involves subjective evaluations of overall environmental comfort and the cognitive assessments and trade-offs related to the potential impacts of behavioral choices (22). Specifically, passengers' perception of the thermal environment is influenced by a combination of factors, including external physical conditions (such as temperature, humidity, and ventilation), individual psychological states, past riding experiences, and expectations regarding the feasibility of behaviors. Under the influence of these factors, the decision-making process regarding thermal adaptation behaviors is both rational and driven by situational and psychological aspects.

Therefore, based on thermal comfort theories and a review of relevant literature, this paper constructs a comprehensive framework to explore the influence of passengers' individual psychological states, perceived thermal comfort (TC), thermal environment expectations, and the presence of fellow passengers on thermal adaptation behaviors. The study aims to conduct an in-depth analysis of bus passengers' adaptive behaviors in thermal environments and their underlying mechanisms from multiple dimensions. The findings will provide valuable recommendations for the design of thermal comfort in buses, thereby enhancing passengers' overall riding experience.

To address the identified research problem, Section 2 of this paper constructs an integrated framework for the thermal adaptation behaviors of public transport passengers, along with the research hypotheses. Section 3 describes the questionnaire design and data collection methods. Section 4, based on the hypothesized model framework, applies Structural Equation Modeling (SEM) to the collected data, validates the proposed framework and research hypotheses, and finally analyzes and discusses the results.

## 2 Conceptual framework and hypothetical model

The theory of planned behavior (TPB) is a foundational framework in environmental behavior research, extensively employed to predict comfort-related and energy-associated behaviors (23). Within TPB, “attitude” denotes an individual's favorable or unfavorable disposition toward performing a specific behavior, encompassing both evaluative judgments and affective responses (24, 25).

**Figure 1:**
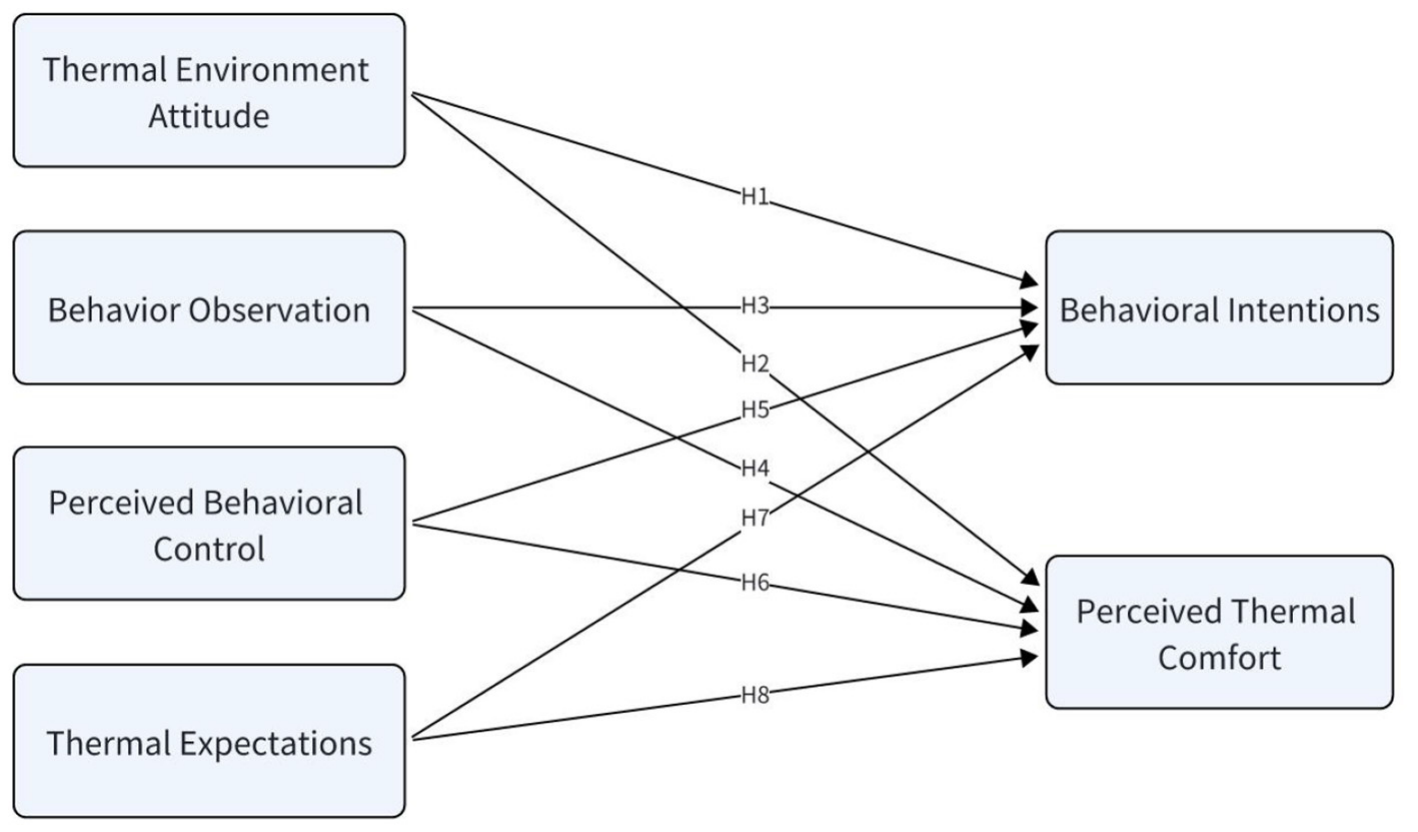
Diagram of the thermal adaptive behavior intention model. The model shows the hypothetical relationship (h1–h9) between key constructs: thermal environment attitude, behavior observation, perceived behavior control, thermal expectation, perceived thermal comfort and behavior intentions.

This study develops a comprehensive model framework by integrating thermal comfort science and social psychology to explore the determinants of thermal comfort and thermal adaptation adjustment behaviors in the riding environment. The structure of the model and hypotheses is shown in Figure 1.

### 2.1 Thermal environment attitude (TEA)

This study conceptualizes passengers' holistic perceptions and affective appraisals of the thermal environment within buses as their “thermal environment attitude,” which integrates evaluations of thermal comfort and propensities for adaptive behavioral adjustments. The research posits that an individual's thermal environment attitude exerts influence on both thermal adaptive behaviors and subjective comfort perceptions. For instance, Valois et al. applied TPB to demonstrate that older adults' thermal environment attitudes significantly predict their heat-adaptive behavioral patterns (26). Richards et al. similarly revealed that passenger comfort is not solely determined by physical environmental inputs but is also modulated by personal attributes, such as travel-related dispositions, which subsequently shape perceived comfort levels (27). Therefore, we propose the following hypotheses:

H1: Thermal environment attitude significantly predicts bus passengers' intentions to engage in thermal adaptation behaviors.

H2: Thermal environment attitude significantly predicts bus passengers' perceived thermal comfort.

### 2.2 Behavior observation (BO)

Social Cognitive Theory (SCT) by Bandura (28) explores the dynamic interaction between individuals, environment, and behavior. Key components include triadic reciprocal determinism, observational learning, and self-efficacy (29). Within SCT, observational learning is how individuals learn and form behavioral intentions (BI) by observing others' behaviors and outcomes. In this study, “behavior observation” means passengers noticing how others on the bus adjust the thermal environment (e.g., opening windows, adjusting clothing). Existing research supports the role of observational learning in behavior within collective environments. Jara-Ettinger et al. (30) noted that in traffic, individuals infer environmental states and adjust their behavior by observing others and speculating intentions. Similarly, on public transport, observing others' environmental adjustments helps passengers perceive the behavior's utility and social acceptability, potentially influencing their own intentions. Therefore, we propose the following hypotheses:

H3: Behavior observation significantly predicts bus passengers' intentions to engage in thermal adaptation behaviors.

H4: Behavior observation significantly predicts bus passengers' perceived thermal comfort.

### 2.3 Perceived behavioral control (PBC)

“Perceived behavioral control” refers to an individual's perception of how easy or difficult it is to perform a specific behavior, reflecting their awareness of factors that may facilitate or hinder the execution of the behavior (23). Perceived behavioral control has a significant predictive effect on behavioral intentions in various behavioral domains. Kalhoro et al. explored the factors influencing environmentally friendly behaviors in the use of urban rail transit in Malaysia and found that perceived behavioral control is an important predictor of public transportation use (31). Hawighorst et al. and Maier et al. by constructing an adaptive comfort model, investigated the factors affecting thermal comfort and found that thermal comfort is related to the level of perceived control (32, 33). In this paper, perceived behavioral control is defined as passengers' subjective assessment of their ability and resources to engage in thermal adaptation behaviors inside the bus. Passengers with higher perceived behavioral control may consider themselves more capable of adjusting the thermal environment inside the bus, thereby improving their own thermal comfort. Therefore, we propose the following hypotheses:

H5: Perceived behavioral control significantly predicts bus passengers' intentions to engage in thermal adaptation adjustment behaviors.

H6: Perceived behavioral control significantly predicts bus passengers' perceived thermal comfort.

### 2.4 Thermal expectations (TE)

In research related to expectations, Barron et al. categorized expectations into four types: ideal, predictive, normative, and unanticipated (34). Schweiker et al. defined the level of thermal expectations in buildings as the alignment between occupants' anticipated thermal experiences and their actual perceived thermal experiences (35). Based on this, this study defines thermal expectations on a bus as the consistency between passengers' anticipated and perceived thermal experiences. An individual's expectations often drive their environmental adjustment behaviors (36). Research shows thermal expectations influence occupants' thermal comfort and satisfaction (37, 38). In the bus context, passengers' thermal expectations may directly affect their perceived thermal comfort and intentions to engage in thermal adaptation behaviors. Therefore, we propose the following hypotheses:

H7: Thermal expectations significantly predict bus passengers' intentions to engage in thermal adaptation behaviors.

H8: Thermal expectations significantly predict bus passengers' perceived thermal comfort.

### 2.5 Perceived thermal comfort (TC)

Perceived thermal comfort refers to passengers' subjective perception and overall evaluation of the thermal environment inside buses, encompassing their comprehensive response to factors such as temperature, humidity, and airflow. Cui et al. discovered in their study that passengers commonly engage in thermal adaptation behaviors, and thermal comfort is closely associated with these adaptive adjustments (39). Perceived thermal comfort serves not only as a crucial metric for evaluating the thermal environment but also as an important factor influencing passengers' intentions to undertake thermal adaptation actions. For example, when passengers perceive a lower level of thermal comfort, they exhibit a stronger propensity to adopt adaptive measures to improve the current thermal environment. Therefore, we propose the following hypotheses:

H9: Perceived thermal comfort can significantly predict the thermal adaptation behavioral intentions of bus passengers.

## 3 Methodology

This study was conducted in the city center of a provincial capital in China, characterized by high traffic density and coverage yet limited subway accessibility. Public buses serve as a primary and frequently used mode of transportation for most adults, especially during commuting hours. As a result, the thermal environment inside buses has become a focus of both public concern and academic research. A retrospective questionnaire-based survey was identified as the most suitable method for this investigation.

By recruiting participants who were actual bus users, the study facilitated the capture of authentic perceptions grounded in real-world experiences, as opposed to those obtained in a controlled laboratory setting. Second, conducting the survey during the summer ensured that thermal sensations were particularly salient due to significantly high temperatures, thereby improving the accuracy and vividness of participants' recall. It is important to note that inherent variables of the bus environment—such as interior temperature, passenger load, and bus arrival/departure times—were not actively controlled. This decision was based on the rationale that manipulating these factors would contradict the study's objective of capturing the actual variations and conditions experienced by users in their daily environments.

### 3.1 Questionnaire design

The questionnaire had two sections. The first section collected demographic and screening information, including age, gender, bus frequency, and travel duration. The second section measured the main study topics: thermal environment attitude, behavioral observation, perceived behavioral control, thermal expectations, perceived thermal comfort, and thermal adaptation behavioral intentions.

All key concepts in the second part were measured using a self-assessment method using a 5 point Likert scale (1 point for “completely disagree” and 5 points for “completely agree”). The specific measurement methods of each construct are as follows:

Thermal environment attitude (TEA): four items are used for measurement, adapted from Fishbein and Ajzen (40) and Valois et al. (26), to evaluate the overall evaluation and emotional judgment of passengers on the bus thermal environment (example item: “I think it is pleasant to provide a comfortable thermal environment on the bus, and it can improve the mood.”)

Behavior observation (BO): four items are used for measurement, which is developed based on Bandura's SCT (28) to assess the frequency of observing other passengers' adaptive behavior through passenger self-report (example item: “when the air in the bus is bad, passengers will open the window.”) Note: this construct measures the perception of others' behavior obtained through self-report, rather than the researchers' direct behavior observation code.

Perceived behavioral control (PBC): four items are used for measurement, adapted from Fishbein and Ajzen (40) and Valois et al. (26), to assess the perceived difficulty of passengers' performing thermal adaptation behavior (example item: “when I feel hot or cold when I take the bus, I can adjust it by changing clothes, opening and closing windows, etc.”)

Thermal expectation (TE): measured by four items, adapted from Rissetto et al. (38), used to assess the consistency between passengers' expected thermal experience and actual perceived thermal experience (example item: “the temperature in the bus always meets my expected needs.”)

Perceived thermal comfort (TC): four items are used for measurement, adapted from Rissetto et al. (38) to capture passengers' subjective evaluation of the thermal environment (example item: “the temperature in the bus is always very satisfactory.”)

Behavioral intention (BI): measured by five items, adapted from Rissetto et al. (38) to assess passengers' willingness to engage in specific thermal adaptation behavior [example item: “if I feel the air in the bus is stuffy, I am willing to adjust my behavior (e.g., open the window).”]

It is noted that this construct measured perceived observations via self-report, not direct external coding of behavior. The complete English version of the questionnaire has been provided as Appendix.

### 3.2 Participants

This study was conducted in Yuelu District, Changsha City during the summer of 2024, focusing on two air-conditioned bus routes (Dakecheng Line 1 and Campus Shuttle) during peak high-temperature periods (12:00–14:00 midday and 17:00–19:00 evening rush hours). The area experiences daily summer temperatures exceeding 30 °C, with limited shading facilities at bus stops resulting in widespread heat exposure for passengers prior to boarding. A total of 373 participants were recruited at bus stops or onboard vehicles. After data quality control following remote survey standards (41), 328 valid responses were retained (149 males and 179 females). Following behavioral research standards for medium effect size (*f*^2^= 0.15), significance level (α = 0.05), and statistical power (0.8) (42), the sample size (*N* = 328) substantially exceeded the minimum required threshold (*N* = 107), ensuring statistical reliability. All surveyed buses were equipped with air conditioning, curtains, and operable windows for thermal regulation. Detailed demographic and sample statistics are shown in the Table 1.

**Table 1:** Descriptive statistical analysis.

**Variant**	**Option**	**Frequency**	**Percentage**
Gender	Male	149	45.40%
	Female	179	54.60%
Age	Under 18 years old	5	1.50%
	18–35 years old	281	85.70%
	36–60 years old	41	12.50%
	Over 60 years old	1	0.30%
Number of bus rides per month	1–3 times	177	54.00%
	4–8 times	86	26.20%
	9–12 times	40	12.20%
	More than 12 times	25	7.60%
The average duration of each bus ride	0–30 min	199	60.70%
	31–60 min	118	36.00%
	More than 60 min	11	3.40%

Data were collected from bus passengers in Changsha, China (N = 328).

### 3.3 Data collection process

The questionnaires were deliberately distributed during both the midday and evening peak hours. This sampling strategy was designed for three key reasons: first, these two periods represent off-peak and busy hours, respectively, enabling comparative analysis between samples; second, it allows for the collection of a more diverse passenger profile (e.g., commuters who mainly travel during the evening peak vs. those traveling at midday); and third, it helps capture a wider range of diurnal temperature variations, with the midday peak representing the highest ambient temperature during the day.

The experimental assistant presented the participants with an informed consent form. The form outlined to the participants the research purpose, experimental methodology, estimated time required for the testing, as well as the compensation for participation in this study. To ensure the privacy of participants, this experiment was conducted anonymously with all data collected being strictly non-identifiable.

(1) Participants chose between scanning a QR code (accessible via any scanner app) or using a paper questionnaire.
(2) They were instructed to follow the instructions and complete items sequentially.
(3) A 10 CNY reward was provided upon survey completion.

### 3.4 Data analysis strategy and evaluation metrics

Data were analyzed using SPSS 23.0 and AMOS 24.0, following a two-stage approach: (1) assessing the measurement model's reliability and validity using Confirmatory Factor Analysis (CFA), which allows for a rigorous test of how well the pre-defined constructs are measured by their respective indicators before examining the structural relationships, and (2) testing the structural model and hypotheses using Structural Equation Modeling (SEM), a comprehensive technique that enables the simultaneous estimation of multiple interrelated dependence relationships and the quantification of unobserved concepts in these relationships. The following evaluation metrics and their widely accepted thresholds were employed (43–45):

Reliability (internal consistency): assessed using Cronbach's alpha (α). This metric measures the extent to which items in a scale are correlated, indicating internal consistency. A value of α ≥ 0.7 is considered acceptable, demonstrating good reliability.
Convergent validity: assessed using Average Variance Extracted (AVE) and Composite Reliability (CR). AVE measures the amount of variance captured by a construct relative to the variance due to measurement error. An AVE >0.5 indicates adequate convergent validity, meaning the construct explains more than half of the variance in its indicators. CR is a measure of the overall reliability of a set of heterogeneous but similar items. A CR >0.7 is considered satisfactory, indicating good internal consistency.
Model fit: chi-square/degrees of freedom (CMIN/DF) between 1 and 3 for excellent fit; Root Mean Square Error of Approximation (RMSEA) < 0.05 for excellent fit and < 0.08 for acceptable fit; Comparative Fit Index (CFI) and Tucker-Lewis Index (TLI) values >0.90 for good model fit. CMIN/DF (χ^2^/df): the normed chi-square. A value between 1 and 3 indicates an excellent fit, while a value up to 5 is acceptable.

Mediating effects were tested using the Bootstrap method (5,000 iterations), with significance assessed via bias-corrected 95% confidence intervals.

## 4 Result

### 4.1 Preliminary analysis

We used SPSS 23.0 and AMOS 24.0 software for data processing and analysis, and used structural equation model (SEM) to verify the hypothesis. Prior to analysis, data quality was ensured through a three-step screening procedure: (i) removing responses with excessively fast completion times; (ii) discarding responses with incorrect answers to reverse-scored items; and (iii) identifying and eliminating responses exhibiting a contradictory response pattern. Specifically regarding the third criterion, a response was considered contradictory if the participant's ratings were consistently negative on all positively-worded items (i.e., scores < 3 on all such items) OR consistently positive on all negatively-worded, reverse-scored items (i.e., scores >3 on all such items). This pattern indicates a lack of attention or failure to comprehend the item phrasing, as established in survey methodology literature (41). Following these criteria, we identified and removed 45 invalid questionnaires.

After data screening, 328 valid responses were analyzed. The skewness values ranged from −1.24 to 0.993, and kurtosis values ranged from −1.027 to 0.088, meeting the assumptions for approximate normality (43, 44). As shown in Table 2, all scales demonstrated excellent internal consistency reliability, with Cronbach's alpha coefficients exceeding 0.84, well above the 0.70 threshold (46).

**Table 2:** Reliability statistics for the study constructs.

**Variable**	**Number of items**	**Cronbach's alpha**
Thermal environment attitude	4	0.852
Behavior observation	3	0.849
Perceived behavioral control	4	0.842
Thermal expectations	4	0.868
Perceived thermal comfort	4	0.866
Behavioral intention	5	0.917

The results show that all variables demonstrated high internal consistency, with Cronbach's alpha values exceeding the recommended threshold of 0.70.

### 4.2 Assessment of the measurement model

**Figure 2:**
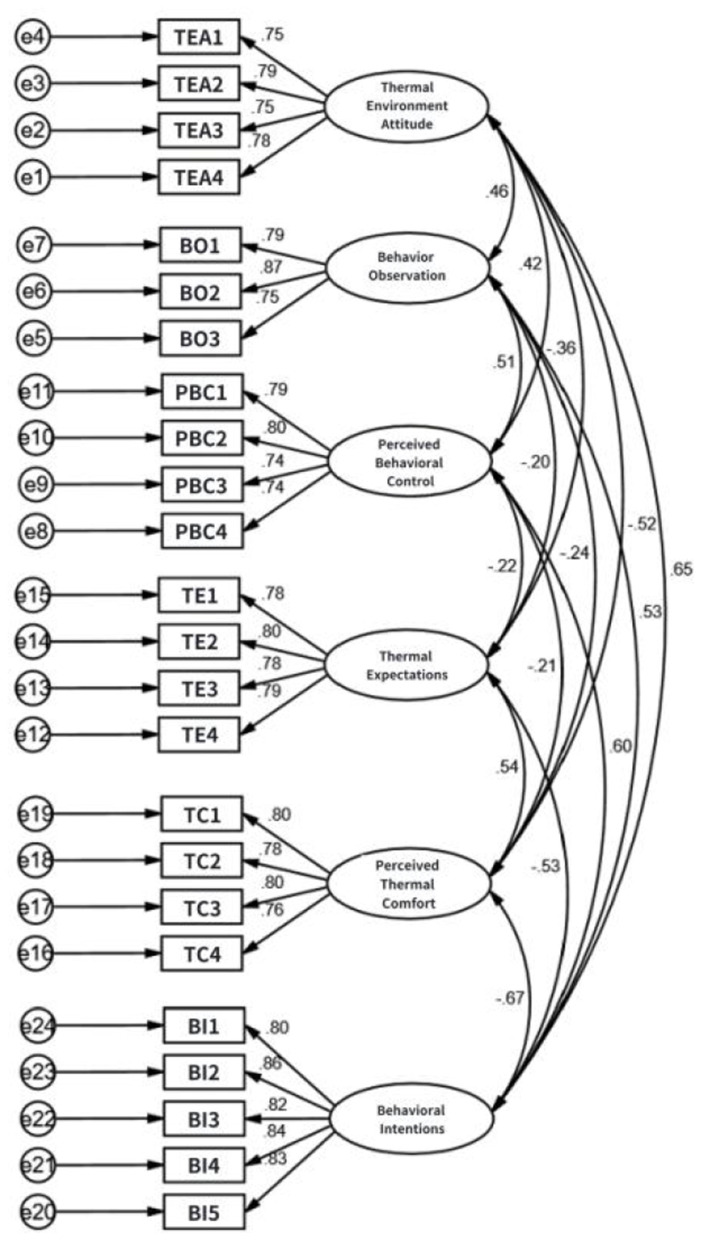
Confirmatory factor analysis model. The model depicts the relationships between the six latent constructs and their respective measurement items. All standardized factor loadings were significant and exceeded 0.70, demonstrating good convergent validity.

The purpose of confirmatory factor analysis (CFA) is to further test the validity of the model, ensuring the correct correspondence between items and dimensions (47). The main aspects examined include construct validity and convergent validity. The analysis model is shown in Figure 2.

According to Table 3, the Confirmatory Factor Analysis (CFA) model showed good fit with the data. Key fit indices were all within acceptable or excellent ranges: CMIN/DF = 1.243, RMSEA = 0.027, and IFI, TLI, and CFI all exceeded the excellent threshold of 0.9.

**Table 3:** Model fit indices for the confirmatory factor analysis (CFA).

**Index**	**Reference standard**	**Consequence**
CMIN/DF	1–3 is outstanding and 3–5 is good	1.243
RMSEA	< 0.05 is outstanding and < 0.08 is good	0.027
IFI	>0.9 is outstanding and >0.8 is good	0.987
TLI	>0.9 is outstanding and >0.8 is good	0.985
CFI	>0.9 is outstanding and >0.8 is good	0.987

All values indicate that the measurement model fits the data well, meeting the recommended thresholds for good model fit.

Convergent validity, which measures how well items within a latent variable correlate, was assessed using Average Variance Extracted (AVE) and Composite Reliability (CR) from the Confirmatory Factor Analysis (CFA). As shown in Table 4, all AVE values were above 0.5 and all CR values were above 0.7, meeting the standards by Fornell and Larcker (45) and Yin (48). This confirms the scales have good convergent validity.

**Table 4:** Assessment of convergent validity.

**Path correlation**	**Estimate**	**AVE**	**CR**
TEA1 < – Thermal Environment Attitude	0.752	0.591	0.852
TEA2 < – Thermal Environment Attitude	0.786		
TEA3 < – Thermal Environment Attitude	0.753		
TEA4 < – Thermal Environment Attitude	0.784		
BO1 < – Behavior Observation	0.791	0.646	0.845
BO2 < – Behavior Observation	0.865		
BO3 < – Behavior Observation	0.751		
PBC1 < – Perceived behavioral control	0.786	0.588	0.851
PBC2 < – Perceived behavioral control	0.796		
PBC3 < – Perceived behavioral control	0.739		
PBC4 < – Perceived behavioral control	0.744		
TE1 < – Thermal expectations	0.784	0.623	0.879
TE2 < – Thermal expectations	0.805		
TE3 < – Thermal expectations	0.782		
TE4 < – Thermal expectations	0.787		
TC1 < – Perceived thermal comfort	0.799	0.618	0.866
TC2 < – Perceived thermal comfort	0.781		
TC3 < – Perceived thermal comfort	0.799		
TC4 < – Perceived thermal comfort	0.764		
BI1 < – Behavioral intention	0.800	0.690	0.918
BI2 < – Behavioral intention	0.862		
BI3 < – Behavioral intention	0.825		
BI4 < – Behavioral intention	0.837		
BI5 < – Behavioral intention	0.828		

Including factor loadings, Average Variance Extracted (AVE), and Composite Reliability (CR). All constructs meet the recommended thresholds (AVE > 0.5, CR > 0.7).

### 4.3 Structural model and hypothesis testing

First, a model fit test was conducted for the thermal adaptive behavior intention model. According to the model fit test results in Table 5, the CMIN/DF (Chi-square to degrees of freedom ratio) = 1.367, which falls within the acceptable range of 1–3. The RMSEA (Root Mean Square Error of Approximation) = 0.033, which is within the excellent range of < 0.05. Additionally, the results for IFI, TLI, and CFI all exceeded the excellent threshold of 0.9. Therefore, based on the above results, it can be concluded that the data collected in this study demonstrates good fit with the established structural equation model.

**Table 5:** Model fit indices for the hypothesized structural model.

**Index**	**Reference standard**	**Consequence**
χ^2^/df	1–3 is outstanding and 3–5 is good	1.367
RMSEA	< 0.05 is outstanding and < 0.08 is good	0.033
IFI	>0.9 is outstanding and >0.8 is good	0.980
TLI	>0.9 is outstanding and >0.8 is good	0.977
CFI	>0.9 is outstanding and >0.8 is good	0.980

The fitting indicators include χ^2^/df, RMSEA, IFI, TLI, and CFI. The results indicate that all indices meeting their respective thresholds for a good fit.

**Figure 3:**
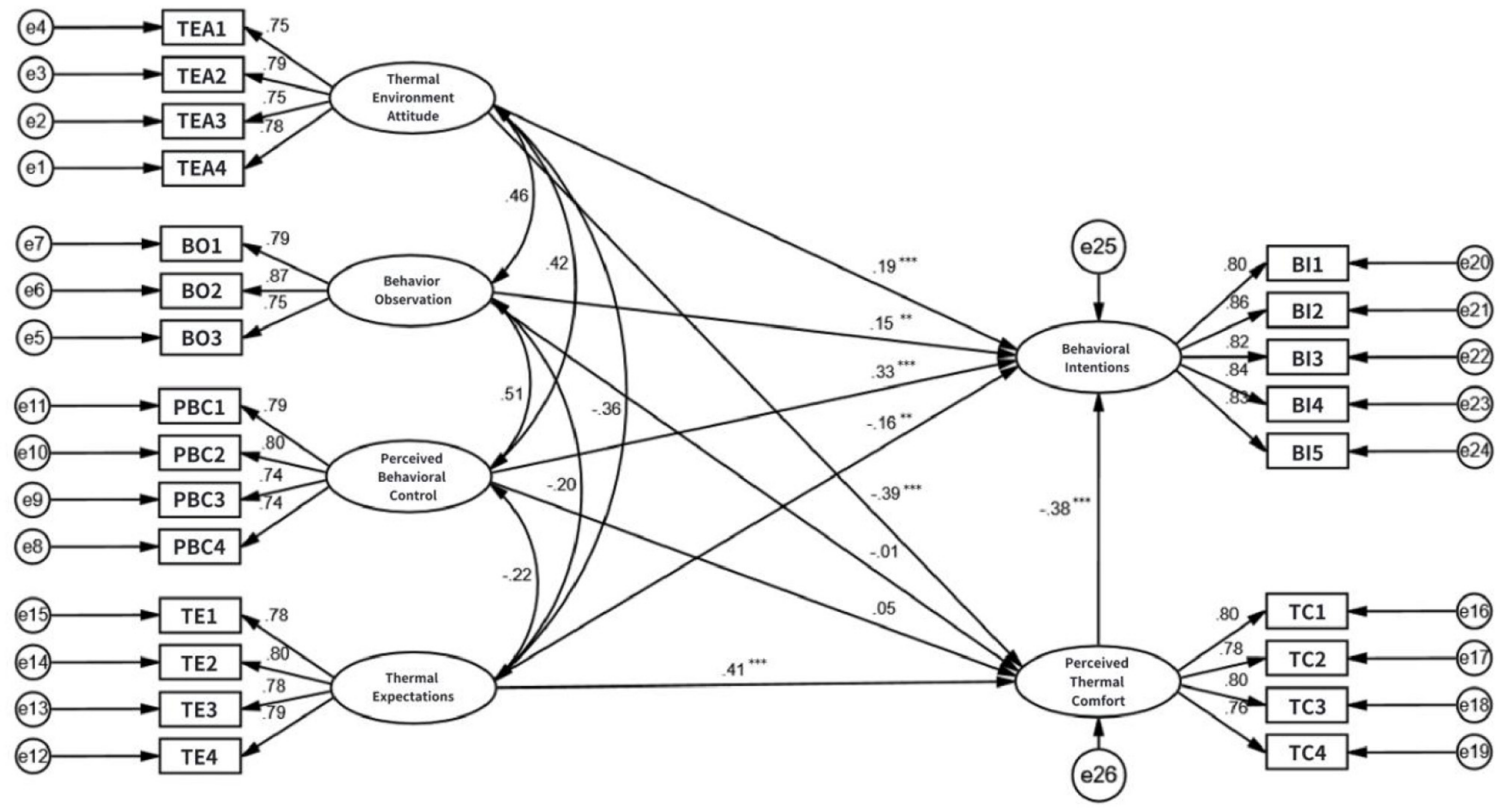
Model and regression coefficients. The model demonstrates good fit to the data (χ^2^/df = 1.367, RMSEA = 0.033, CFI = 0.980, TLI = 0.977). Solid lines represent significant paths (**p < 0.01, ***p < 0.001), while dashed lines indicate non-significant paths (n.s.).

Based on the acceptable model fit, the paths in the constructed structural equation model were tested. The results are shown in Table 6, and the SEM model results are presented in Figure 3.

**Table 6:** Hypothesis testing results for the structural model pathways.

**Hypothesis**	**Pathway**	**Non-standardized coefficient**	**Standardized coefficient**	**S.E**.	**C.R**.	***p*-value**
H1	Behavioral intention < – Thermal environment attitude	0.217	0.194	0.061	3.535	^***^
H2	Perceived thermal comfort < – Thermal environment attitude	−0.464	−0.385	0.087	−5.354	^***^
H3	Behavioral intention < – Behavior observation	0.157	0.152	0.052	3.025	0.002^**^
H4	Perceived thermal comfort < – Behavior observation	−0.008	−0.007	0.077	−0.106	0.916
H5	Behavioral intention < – perceived behavioral control	0.325	0.325	0.053	6.167	^***^
H6	Perceived thermal comfort < – Perceived behavioral control	0.049	0.046	0.073	0.672	0.502
H7	Behavioral intention < – thermal expectations	−0.173	−0.157	0.053	−3.263	0.001^**^
H8	Perceived thermal comfort < – Thermal expectations	0.482	0.407	0.074	6.494	^***^
H9	Behavioral intention < – perceived thermal comfort	−0.354	−0.381	0.053	−6.630	^***^

The table shows the standardized path coefficients, standard errors, critical ratios, and p-values. Results indicate that the majority of the proposed hypotheses were supported by the data. ^**^p < 0.01, ^***^p < 0.001.

**Figure 4:**
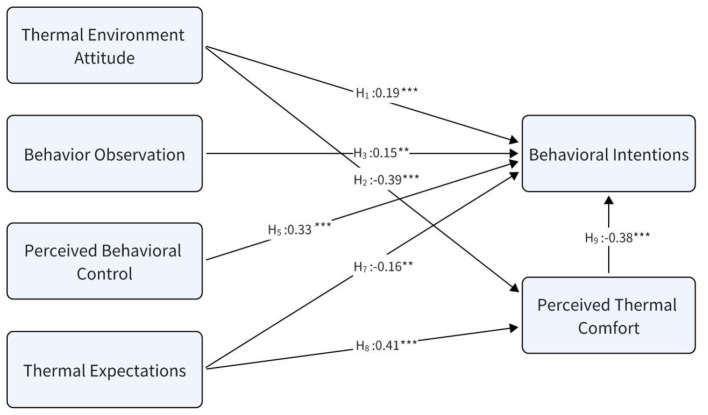
The final structural model of passengers' thermal adaptation behavioral intentions. The model displays only the significant paths, highlighting perceived behavioral control (PBC) and thermal environment attitude (TEA) as the primary drivers of intention, with perceived thermal comfort (TC) acting as a key mediator (**p < 0.01, ***p < 0.001).

The analysis revealed that thermal environment attitude, behavioral observation, and perceived behavioral control all significantly and positively influenced passengers' thermal adaptation behavioral intentions (supporting H1, H3, and H5). Regarding perceived thermal comfort, attitude had a significant negative effect (supporting H2), but observation and perceived control did not show a significant influence (H4, H6 not supported). This suggests perceived comfort isn't significantly affected by observing others' behaviors or one's own sense of control. Furthermore, both thermal expectations and perceived thermal comfort significantly negatively influenced adaptation intentions (supporting H7, H9). Thermal expectations also had a significant positive effect on perceived thermal comfort (supporting H8). Figure 4 illustrates the final model.

### 4.4 Mediating effect analysis

Mediating effects were tested using the Bootstrap method (5,000 iterations), with significance assessed via bias-corrected 95% confidence intervals (significant if the interval does not contain zero) (49). Results indicated that perceived thermal comfort had a significant mediating effect between passengers' attitudes and behavioral intentions (β = 0.147, *p* < 0.001), as well as between thermal expectations and behavioral intentions (β = −0.155, *p* < 0.001). Specific results are shown in Table 7.

**Table 7:** Mediation analysis results using the bootstrap method.

**Indirect effect tested**	**Total effects**		**Direct effects**		**Indirect effects**		**Indirect effect-Bias-corrected 95%CI**	
	β	***p*** **value**	β	***p*** **value**	β	***p*** **value**	**Lower**	**Upper**
TEA → PTC → BI	0.341	0.000	0.194	0.004	0.147	0.000	0.088	0.274
TE → PTC → BI	−0.312	0.000	−0.157	0.005	−0.155	0.000	−0.293	−0.082

The results confirm the significant mediating role of perceived thermal comfort (TC) in the relationships between thermal environment attitude (TEA), thermal expectations (TE), and behavioral intention (BI).

### 4.5 Differences among demographic characteristics

The differences between demographic characteristics were analyzed using *t*-tests, and the results are shown in the Table 8. There were no significant gender differences in thermal environment attitude, behavioral observation, and perceived behavior. However, significant gender differences were found in thermal expectations, perceived thermal comfort, and behavioral intentions (*t* = 2.287, *p* < 0.05; *t* = 4.927, *p* < 0.001; *t* = −3.233, *p* < 0.01).

**Table 8:** Differences among demographic characteristics.

**Dimension**	**Male**	**Female**	***t*-value**	**Significance**
TEA	3.9748 ± 0.75211	4.1103 ± 0.63271	−1.745	0.082
BO	3.5906 ± 0.79234	3.6499 ± 0.76133	−0.687	0.493
PBC	3.4446 ± 0.78513	3.5684 ± 0.78019	−1.426	0.155
TE	2.6611 ± 0.83932	2.4665 ± 0.67061	2.287	0.023
TC	2.8171 ± 0.90496	2.3799 ± 0.65253	4.927	< 0.001
BI	3.5047 ± 0.92286	3.8078 ± 0.74181	−3.233	0.001

Results of independent samples t-tests examining gender differences in the study variables. Significant differences were found between male and female passengers in thermal expectations (TE), perceived thermal comfort (TC), and behavioral intention (BI).

## 5 Discussion

### 5.1 Impact of thermal comfort on thermal adaptation behavior

Perceived thermal comfort, as a core variable, not only directly influences thermal adaptation behavioral intentions but also acts as a mediator between other key variables. The results show that the significant negative path coefficient (β = −0.381) reveals a clear logical relationship: a lower level of perceived thermal comfort (i.e., a greater state of discomfort) directly predicts a stronger intention among passengers to engage in thermal adaptation behaviors intentions. This aligns with the core principle of the adaptive thermal comfort model proposed by De Dear (8), which posits that individuals regulate their behavior (e.g., changing the environment, adjusting clothing, or changing position) in uncomfortable conditions to improve thermal comfort. This finding directly supports Hypothesis H9, which was based on this very principle. This discovery is particularly important in subtropical cities like Changsha. The hot, humid summers and cold, damp winters exacerbate passengers' thermal discomfort, prompting them to more frequently form intentions to engage in thermal adaptation behaviors within the bus cabin. Based on these findings, bus operators should optimize thermal environment management by implementing measures such as dynamic temperature control and personalized adjustment tools to enhance passenger thermal comfort. For example, installing sensors inside buses to monitor temperature, humidity, and passenger density would enable dynamic adjustments of air conditioning temperature and ventilation intensity based on environmental changes and crowd density (50). Furthermore, future bus designs could divide the interior into different temperature zones, allowing passengers to choose seats based on their individual thermal comfort preferences (51).

Interestingly, the analysis of the thermal comfort mediation effect revealed that no significant direct effects of behavioral observation or perceived behavioral control on perceived thermal comfort. We propose two possible explanations for this phenomenon:

(1) This is consistent with the traditional understanding of thermal comfort as defined by ASHRAE and used in Fanger's PMV model (6), which emphasizes environmental factors and individual physiological responses. Passengers' perceived thermal comfort is primarily influenced by the thermal environment of the bus ride and their individual thermal sensations, rather than by the behavior of surrounding passengers or others in the environment.
(2) Perceived behavioral control, as a construct within the theory of planned behavior (23), primarily emphasizes the conditions under which behavior occurs, and it does not have a direct relationship with passengers' perceived thermal comfort. Perceived behavioral control has a greater influence on behavioral intentions rather than on immediate feelings of comfort. These results are consistent with the findings of Zhou et al. (52), who emphasized that in enclosed environments like buses, radiant temperature and air temperature are the most critical environmental factors affecting passengers' thermal comfort.

This result suggests that improving passengers' perceived thermal comfort requires focusing on measures that directly enhance the environment, rather than relying on indirect influences through perceived behavioral control or behavioral observation.

### 5.2 The dual pathways of thermal environment attitude influence

Thermal environment attitude is an important variable influencing thermal adaptation behavioral intentions, and its impact is confirmed through two distinct pathways, supporting both H1 and H2.

The results show a significant positive direct effect of thermal environment attitude on thermal adaptation behavioral intentions (β = 0.194, supporting H1). This finding is firmly grounded in the core principles of the theory of planned behavior (TPB), which posits attitude as a proximal determinant of behavioral intention (23, 40). This implies that when passengers internally recognize the value of a comfortable thermal environment and hold positive evaluations toward it, this attitude itself constitutes a powerful internal driver. This drive can be directly translated into the willingness to undertake adaptive behaviors (such as adjusting clothing or operating windows), meaning that such value-based proactive actions might occur even when the immediate thermal sensation is not intolerable. Consequently, this direct pathway highlights the fundamental importance of fostering a positive TEA—it can make adaptive behavior a spontaneous, intrinsically motivated process.

Furthermore, a significant negative effect was found between thermal environment attitude and perceived thermal comfort (β = −0.385, supporting H2). This finding, seemingly counterintuitive at first glance, actually reveals a deeper, indirect psychological mechanism through which attitude influences behavior. We interpret this phenomenon as follows: passengers with a more positive TEA likely establish a higher or more defined “comfort standard” or “expectation benchmark” in their minds (35, 53). This elevated standard makes their assessment of the actual thermal environment more critical and their sensitivity to any subtle discomfort deviating from their ideal state heightened. In other words, they are not objectively in a worse environment; rather, due to their enhanced cognitive attention and evaluative sensitivity, they are more inclined to rate the existing environment as “uncomfortable.” This mechanism expands the role of TEA from a simple “driver” to a “sensitizing agent.” It indirectly drives behavioral intention by lowering the individual's perceived comfort level (TEA → TC), thereby activating the pathway where discomfort triggers behavioral intention (TC → BI).

Consequently, as hypothesized, the influence of thermal environment attitude on behavioral intentions is also partially mediated by perceived thermal comfort (TEA → TC: β = −0.385; TC → BI: β = −0.381). These findings are consistent with the theory of planned behavior, which posits that attitude is a key predictor of behavioral intention (40, 54). The direct effect demonstrates that a positive attitude toward the thermal environment can independently motivate individuals to form thermal adaptation intentions. The mediating role of perceived thermal comfort, on the other hand, reveals another pathway through which attitude exerts its influence. Individuals with a positive thermal environment attitude are likely to place greater importance on thermal comfort (53). This increased importance may lead them to be more attentive to their thermal sensations, thereby responding more proactively to any perceived discomfort. This responsiveness, in turn, strengthens the link between perceived thermal comfort and their intentions to engage in adaptive behaviors. This dual-pathway model thus enriches the TPB framework by introducing a crucial perceptual-affective mechanism: a positive TEA not only directly fuels the intention to act but also sharpens one's sensory evaluation of the environment, creating a more potent, discomfort-driven impetus for adaptation. It underscores that attitude can influence behavior not just by making an action seem desirable, but also by making the status quo feel less acceptable.

The findings suggest that promoting and educating passengers to develop positive thermal environment attitudes can effectively enhance their willingness to address thermal environment issues, both directly and indirectly. A positive thermal environment attitude among passengers can directly motivate the formation of their thermal adaptation behavioral intentions. Additionally, it can indirectly influence intentions by increasing passengers' attention to their own thermal comfort, making them more likely to take action when they perceive a discrepancy between their desired and actual thermal states. Therefore, public awareness campaigns could highlight the importance of a comfortable thermal environment not only for overall travel satisfaction but also for personal wellbeing and proactive health management, thereby cultivating a foundational positive attitude. This is particularly important for older adults, as their ability to perceive the thermal environment may decline due to physiological conditions (such as decreased thermoregulatory capacity) (55). It is necessary to provide external support on buses to raise their awareness of the thermal environment. For example, thermal environment awareness campaigns could be conducted in communities and public transit areas targeting older adults, explaining the potential health risks of extreme heat or cold weather. Additionally, providing real-time visual feedback on the current thermal environment inside the bus can help them stay informed about the conditions, thus supporting their formation of thermal adaptation intentions. The practical implication extends beyond physical adjustments to the environment; it calls for targeted communication strategies that shape passenger attitudes and compensate for varying levels of thermal sensitivity, ensuring that all passengers are empowered and motivated to take adaptive actions for their own comfort.

### 5.3 The influence of perceived behavioral control

Perceived behavioral control is an important factor reflecting an individual's regulatory ability, and it has a significant positive direct effect on thermal adaptation behavioral intentions (β = 0.325). Perceived behavioral control is defined as an individual's assessment of their ability to take action within a given environment. This aligns with the theory of planned behavior's proposition that perceived behavioral control directly influences behavioral intentions (23, 32), thus supporting Hypothesis H5. This result indicates that passengers' thermal adaptation intentions depend not only on the external environment but also on the operability and effectiveness of onboard facilities. When adjustment devices are easy to use, resources are sufficient, and their distribution is reasonable, passengers' sense of control is significantly enhanced (38), which in turn increases their thermal adaptation intentions. For example, providing more adjustment resources on buses (such as zoned air conditioning and adjustable air vents) and posting clear and easy-to-understand instructions for the equipment can improve passengers' operability and sense of control, ultimately promoting their thermal adaptation intentions.

### 5.4 The influences of thermal expectations and behavioral observation

Passengers' thermal expectations have a direct negative effect on their behavioral intentions (β = −0.157, supporting H7). When the thermal environment inside the bus aligns with their expectations, passengers tend to maintain the status quo, reducing their intentions to engage in thermal adaptation behaviors. The study also found a significant indirect effect of thermal expectations on behavioral intentions through perceived thermal comfort. This mediating pathway (TE → TC → BI) is substantiated because thermal expectations significantly predict perceived thermal comfort (thus supporting H8, β = 0.407), and perceived thermal comfort, in turn, significantly predicts behavioral intentions (β = −0.381). These findings support previous research indicating that thermal expectations play an important role in shaping thermal comfort perceptions and subsequent behavioral intentions (35, 37). The results suggest that thermal expectations act as a reference point, influencing individuals' perceptions and behavioral decisions regarding the current thermal environment, and consequently, their behavioral intentions (56). To address this, buses need to offer more flexible thermal comfort adjustment mechanisms to meet passengers' individual needs. For example, establishing real-time feedback channels (such as in-bus screens or mobile apps) would allow passengers to directly submit their opinions on the temperature and comfort level inside the bus, helping the system dynamically adjust the environment based on feedback and promptly respond to passengers' expectations, thereby potentially mitigating the need for extensive individual thermal adaptation and improving overall comfort.

Behavioral observation demonstrates a significant yet distinct pathway in shaping thermal adaptation behavioral intentions (β = 0.152, supporting H3). While the effect size is relatively modest compared to other predictors, this finding substantiates the role of observational learning in shared thermal environments, as postulated by Social Cognitive Theory (28, 57). The mechanism operates through dual processes: witnessing fellow passengers' adaptive behaviors (such as adjusting clothing, operating windows, or using personal cooling devices) provides both informational cues about effective adaptation strategies and normative signals about socially acceptable actions within the specific context of public transportation. The relatively smaller effect size can be understood through the unique characteristics of bus environments. As transient social spaces where passengers maintain varying degrees of social distance, the observational learning process may be moderated by factors such as journey duration, passenger density, and cultural norms regarding social monitoring. This suggests that while social learning undoubtedly occurs, its potency may be constrained by the specific contextual features of public transit settings. These insights point to strategic opportunities for environmental design that can optimize the social learning potential in bus cabins. Visibility-enhanced seating arrangements that maximize visual access to common adaptation behaviors, coupled with discreet informational signage demonstrating recommended adaptation techniques, can create an environment conducive to positive social learning. Furthermore, the strategic placement of clearly operable controls and adjustable features can serve as both functional elements and visible prompts for adaptation behaviors.

The confirmation of this social pathway, operating alongside the established comfort-driven and expectation-based pathways, underscores the multifaceted nature of behavioral adaptation in public transit environments. This understanding advocates for integrated intervention approaches that simultaneously address physical comfort parameters while harnessing the potential of social influence mechanisms to promote adaptive behaviors, ultimately contributing to enhanced passenger comfort through multiple complementary channels.

### 5.5 Differences among demographic characteristics

Research results reveal significant gender differences in thermal expectations (*t* = 2.287, *p* < 0.05), perceived thermal comfort (*t* = 4.93, *p* < 0.01), and behavioral intentions (*t* = −3.23, *p* < 0.001) (58). Women rated the bus's thermal environment as less meeting their expectations than men, indicating lower satisfaction and a greater gap between actual and expected experiences. Women also reported significantly lower perceived thermal comfort, feeling generally more uncomfortable (58). This may be due to physiological differences like women's higher sensitivity to temperature/humidity and lower metabolism (59).

Furthermore, female passengers scored higher than males in thermal adaptation behavioral intentions, demonstrating a stronger willingness to take proactive measures to improve their thermal comfort and maintain thermal balance. Therefore, public transportation thermal environment management should fully consider gender differences and provide more female-friendly adjustment facilities, such as establishing women-only areas or using more ventilated and breathable materials in seating areas, to enhance their riding comfort.

## 6 Conclusion

This study contributes to the theoretical framework from multiple perspectives. First, by integrating the theory of planned behavior, Social Cognitive Theory, and theories related to thermal comfort, a comprehensive model was developed to systematically analyze the effects of thermal environment attitude, perceived behavioral control, thermal expectations, behavioral observation, and perceived thermal comfort on passengers' thermal adaptation behavioral intentions. The research broadens the perspective of the existing field of thermal adaptation behavior and, through cross-theoretical integration and innovation, provides a more comprehensive and systematic theoretical foundation for studies on thermal environment behavior. The main conclusions of the study are as follows:

1. Perceived thermal comfort is a core variable influencing passengers' thermal adaptation behavioral intentions, and it also serves as a mediating variable between thermal environment attitude, thermal expectations and behavioral intentions. The study found that individuals with a positive thermal environment attitude place greater importance on thermal comfort, thereby responding more proactively to any perceived thermal discomfort. On the other hand, thermal expectations have a significant positive effect on perceived thermal comfort, indicating that the more the bus's thermal environment aligns with passengers' expectations, the stronger their sense of comfort; when expectations are not met, passengers are more inclined to engage in thermal adaptation behaviors.
2. Thermal environment attitude and perceived behavioral control demonstrate distinct yet complementary pathways in influencing passengers' thermal adaptation behavioral intentions. The study reveals that a positive thermal environment attitude functions through a dual mechanism: it serves as a direct motivator for behavioral intentions while simultaneously acting as an indirect sensitizer that amplifies discomfort perception, thereby enhancing adaptation intentions through increased thermal awareness. Meanwhile, perceived behavioral control emerges as the strongest direct predictor (β = 0.325) of behavioral intentions, underscoring passengers' reliance on actionable adaptation options. This significant impact highlights the crucial importance of operational simplicity and accessibility in onboard facility design. The clear functional separation between these psychological pathways—where attitude operates through evaluative-emotional channels while perceived behavioral control functions through efficacy-assessment channels—provides a more nuanced understanding of how different cognitive and affective processes collectively shape behavioral outcomes in thermal adaptation contexts. This distinction not only clarifies the operational mechanisms of these psychological factors but also suggests the need for differentiated intervention strategies targeting attitude formation vs. control enhancement in thermal environment management.
3. Behavioral observation significantly impacts thermal adaptation behavioral intentions. The significant effect of behavioral observation (β = 0.152) confirms that thermal adaptation decisions are socially embedded processes. This finding expands traditional individual-focused thermal comfort models by incorporating social learning mechanisms, particularly relevant in the context of public transportation where passengers continuously observe and learn from each other's adaptive behaviors. Passengers' thermal adaptation behavior is, to some extent, influenced by the behavior of others.

## 7 Limitations and future work

Conducted in Changsha, China (a humid subtropical city), this study's findings may have limited applicability due to significant climate variations (e.g., temperature, humidity) and differences in bus services (e.g., AC availability) across cities. Future research should compare passengers' thermal adaptation behaviors under a wider range of conditions to develop more universally applicable solutions.

Furthermore, a limitation was the lack of strict control over environmental variables (e.g., temperature, wind speed, humidity, clothing insulation) during data collection, which may have influenced the results. For example, uncontrolled conditions could cause differences in perceived thermal comfort, affecting the measurement of behavioral intentions. Future research should use controlled laboratory settings or real-time environmental monitoring inside and outside the bus. Analyzing psychological, thermal, and physiological data together can more precisely assess the impact of variables on adaptation behaviors.

## Data Availability

The raw data supporting the conclusions of this article will be made available by the authors, without undue reservation.
