# Supplementary material for: Study on thermal adaptation behaviors of bus passengers
Source: Front Public Health. 2025 Nov 28;13:1707181. doi: 10.3389/fpubh.2025.1707181 (PMC12698646; doi:10.3389/fpubh.2025.1707181)
Supplement: Supplementary file 1 [file Table_1.docx]

Supplementary Material

1. **Appendix**

This appendix provides the English translations of the questionnaires used in the study. The questionnaire measures six primary constructs (TEA, BO, PBC, TE, TC, BI) using a total of 25 items on a five-point Likert scale.

| Construct | ID | Question content （Strongly disagree (1)/Strongly agree (5)） | Reference source |
| --- | --- | --- | --- |
| Thermal environment attitude (TEA) | 1 | I think that providing a comfortable thermal environment on buses is pleasant and enhances one's mood. | [26], [40] |
|  | 2 | I think that providing a comfortable thermal environment on buses is unimportant. (Reverse-coded item). |  |
|  | 3 | I think that the thermal environment on the bus greatly affects my riding experience. |  |
|  | 4 | I think that a stable thermal environment on the bus is reassuring. |  |
| Behavior observation (BO) | 1 | Passengers traveling on the bus will open the windows when the air is poor | [28] |
|  | 2 | Passengers traveling on the bus will adjust the thickness of their clothing when the temperature is not high |  |
|  | 3 | My friend who was traveling on the bus complained to me about the discomfort of the temperature inside the car |  |
|  | 4 | Passengers traveling on the bus will close the curtains when the sunlight inside the bus is strong |  |
| Perceived behavioral control (PBC) | 1 | When I feel hot or cold while taking the bus, I adjust my clothes, open and close windows, etc | [26], [40] |
|  | 2 | When I feel stuffy and hot while taking the bus, I always manage to improve my discomfort by adjusting my clothes, opening and closing windows, etc |  |
|  | 3 | When I feel dry while taking the bus, I can always improve my discomfort by drinking water, etc |  |
|  | 4 | When taking the bus, when I am exposed to strong sunlight, I can always improve my discomfort by adjusting curtains, changing positions, etc |  |
| Thermal expectations(TE) | 1 | The temperature inside the bus always meets my expected needs | [38] |
|  | 2 | The air conditioning cooling/heating effect of buses is always satisfactory |  |
|  | 3 | The air quality inside the bus always meets my expected needs |  |
|  | 4 | The humidity inside the bus always meets my expected needs |  |
| Perceived thermal comfort (TC) | 1 | The air quality inside the bus always feels fresh and comfortable | [38] |
|  | 2 | The humidity inside the bus is always comfortable |  |
|  | 3 | The temperature inside the bus is always very satisfactory |  |
|  | 4 | The sunshine inside the bus is always very comfortable |  |
| Behavioral intention (BI) | 1 | If I feel the air inside the bus is stuffy, I am willing to make behavioral adjustments (e.g., opening the window, etc.). | [38] |
|  | 2 | If I feel the temperature inside the bus is too hot or too cold, I am willing to make behavioral adjustments (e.g., adjusting the thickness of my clothing, etc.). |  |
|  | 3 | If direct sunlight inside the bus makes me uncomfortable, I am willing to make behavioral adjustments (e.g., adjusting the curtains, etc.). |  |
|  | 4 | If there is strong airflow at my seat, I am willing to make behavioral adjustments (e.g., changing seats, etc.). |  |
|  | 5 | If I feel uncomfortable with the temperature inside the bus, I am willing to make behavioral adjustments (e.g., adjusting the air conditioning temperature, etc.). |  |
